# Assessing antifeminism introducing the *Leipzig Antifeminism Short Scale* (LAF-S) in a German representative sample

**DOI:** 10.3389/fpsyg.2025.1596397

**Published:** 2025-12-03

**Authors:** Charlotte Höcker, Ayline Heller, Elmar Brähler, Oliver Decker

**Affiliations:** 1 Department of Psychosomatic Medicine and Psychotherapy, University Medical Center, Leipzig, Germany; 2 Leipzig University, Leipzig, Germany; 3 Survey Design and Methodology, GESIS – Leibniz Institute for the Social Sciences, Mannheim, Germany; 4 Department of Psychosomatic Medicine and Psychotherapy, University Medical Center of the Johannes Gutenberg University Mainz, Mainz, Germany; 5 Sigmund Freud University Berlin, Berlin, Germany

**Keywords:** antifeminism, validation, scale construction, measurement invariance, latent means

## Abstract

While gender equality and attitudes towards gender roles have become a topic of increased public interest, little attention has been drawn to the phenomenon of *antifeminism* as a distinct form of opposition against women’s emancipation. Although some approaches to measuring antifeminism exist, no instrument has yet been tested and validated on a large scale. The present study examines the psychometric properties of the four-item *Leipzig Antifeminism Short Scale* (LAF-S) in a representative German sample (*N* = 2,459). Results suggest good internal consistency and external validity. Unidimensionality was confirmed, and measurement invariance may be assumed across various sociodemographic groups. Differences in latent means were analyzed and discussed. The LAF-S thus proved to be a valuable and highly efficient instrument for measuring antifeminism on a large scale. Future studies should aim to validate the scale in different cultural settings, using the translation provided here.

## 1 Introduction

In June 2022, we have witnessed a historic regression of previously hard-won women’s rights in the USA. “Roe v. Wade,” a famous precedent that since 1973 has guaranteed women the privacy to decide for themselves whether to terminate or continue a pregnancy without the interference of the state, was overturned by a new Supreme Court decision.

The right and its fightback have been the subject of longstanding antifeminist mobilizations. Cicerchia (2022) speaks of the end of Roe as “being the story of the most organized, militant, and successful conservative social movement of the past 50 years.” In the political success of the right-wing minority (at 70 per cent, a clear majority of US citizens support a right to abortion), she also sees a failure of the left, the Democratic Party, non-profit reproductive rights and social justice organizations, and the feminist movement. In order to discuss the question of what to learn from current developments in the US for democratic debates all over the world, it is central to address the ideology that led to such a passionate and successful right-wing attempt at hegemony in the debate on the current amendments to the law: Antifeminism.

The feminist demand for women’s sexual self-determination and for reproductive rights becomes a threat to the right’s population policies, which in the German context can be described as “völkisch^1^” and showing clear parallels to American White nationalism. Hatred of women and feminists as well as attacks on their emancipation achievements are all firmly anchored in the argumentations and mobilization strategies of right-wing extremist actors and parties, with a historical tradition and currently particularly evident in right-wing terrorism (Blum, 2025; Autor*innenkollektiv FE.IN, 2019). However, antifeminist ideologies are also connectable to the broad population (Küpper, 2018). After receiving little attention for years, antifeminism has started to become more of a topic in social psychological research on right-wing extremism and authoritarianism in the past years (Höcker et al., 2020; Kalkstein et al., 2024). Yet, measures to investigate antifeminist attitudes in a general population are still scarce and there is no short measurement instrument to monitor antifeminism on a large scale so far. We want to address this gap by introducing and validating a new, economical instrument, the *Leipzig Antifeminism Short Scale* (LAF-S) for the efficient measurement of antifeminism in large-scale survey contexts.

### 1.1 Theoretical considerations

While *feminism* is understood as an emancipation movement, aiming at the abolition of the marginalization and oppression of women, and enforcing the democratization of gender relations and the critique of male domination, *antifeminism* may be defined as an organized opposition to feminist emancipation efforts. Henninger et al. (2021), following Birsl (2020) and Lang and Fritzsche (2018), highlight the ideological position of antifeminism, “which is concerned with the opposition to […] processes of sociopolitical liberalization and denormalization of gender relations, as well as with the maintenance of heteronormative relations of domination” (Lang and Fritzsche, 2018, p. 340, translated by the authors).

For Birsl (2020), the field of action of feminist emancipation efforts is not limited to the hierarchical order of bipolar gender stereotypes, but rather it permeates all social conditions. She identifies antifeminism as a “thin ideology” with an antisemitic bias: it is based on a juxtaposition of progressive efforts to address women’s and LGBTI* concerns, and on the preservation of the heteronormative family as the “primordial cell” of society in neoliberalism.

Antifeminism, like modern antisemitism, emerged in the 19th century as an antimodernist resentment specifically directed against the egalitarian promises and contradictory dynamics of liberal and pluralistic modernity (Volkov, 2001; Hessel and Misiewicz, 2020). Feminists are personified as guilty of fundamental, contradictory changes in gender relations and families in the course of modernity. The term *antifeminism* was defined in a essay by Dohm (1902), who also interpreted it in the context of the rise of modern antisemitism. A key difference has been pointed out though (Hessel and Misiewicz, 2020): Unlike antisemitism, the rejection of women’s emancipation was almost universally shared and hardly recognized, thus fulfilling an important mobilizing and bridging function between the bourgeois and conservative camps. According to historian Planert (1998), antifeminism, characterized by its opposition to feminist movements, can be distinguished both from sexism, a term focusing on gender-based discrimination, and from misogyny, a pervasive devaluation of women deeply entrenched in societies.

When looking at these analyses and definitions of antifeminism, clear connections to the concept of (right-wing) authoritarianism arise. Modern theories of authoritarianism typically distinguish three dimensions: authoritarian aggression, authoritarian submission, and conventionalism (Altemeyer, 1981, 1996). The *authoritarian syndrome* may be defined as a person’s affinity for rigid ideologies that “allow them to submit to an authority, to share in its power, and to call for others to be devalued in the name of the system” (Decker et al., 2022, p.28). Antifeminist, misogynist, and sexist acts can thus all serve to fulfill an authoritarian need and establish a clear moral distinction between “good” and “bad” behavior (Manne, 2019, p. 146). Moreover, by humiliating women and trying to assign them a certain role, hierarchical power relations can be stabilized. We make use of this connection between antifeminism and authoritarian and right-wing extremist attitudes in the validation process, expecting them to be related, and yet distinct phenomena.

The major focus of German and Austrian antifeminism research is the study of organized antifeminist actors. However, antifeminism can manifest in many different ways. Some forms remain almost unnoticed as they manifest in inconspicuous phenomena, such as the non-perception of the everyday oppression and marginalization of women, the devaluation and segregation of the “female sphere” (e.g., in the low social status of “women’s stuff” and reproductive work), the defense of the traditional gender order against feminist interests, and the rejection of the “feminization” of society. One main aim of this study is to provide a measurement instrument to monitor antifeminist attitudes in the general population, thus extending current research by enabling the investigation of antifeminism in all social strata.

### 1.2 Existing scales and empirical findings

While a lot of scales exist to measure gender role sexism in general population samples, only little research has been conducted on antifeminism specifically. We conceptualize these two constructs to be very close, but distinct, as antifeminism goes beyond attitudes towards parenthood or the division of labor, addressing a general opposition to the emancipation of women in many different spheres. However, it makes sense to take a look at existing scales on gender role sexism and the results they produced to place antifeminism in a nomological network and make predictions about the behavior of our scale when it comes to sociodemographic variables, e.g., sex, place of residence in East and West Germany, educations, age, income and religious affiliation.

The most commonly used measure to assess gender role attitudes is the *Gender-role attitudes* (ISSP 94; Braun, 1999). Consisting of eleven items on three dimensions, it was first used in the International Social Survey Programme (ISSP) in 1994 and administered in 24 countries. 4-6-item versions of the scale had been used before in the American General Social Survey (GSS), the German General Social Survey (ALLBUS), the British Social Attitudes Survey as well as the Polish General Social Survey (Braun, 1999). The scale focuses on women’s participation in the labor market and traditional vs. egalitarian family structures and it does not specifically address the issue of antifeminism (Walter, 2018, pp. 47). Some interesting observations regarding sociodemographic groups were made though: as expected, women generally hold more egalitarian attitudes than men. More egalitarian views on the division of labor were also prevalent in the former eastern compared to the western German states (Baier, 2014). This was ascribed to the working policies of the former socialist German Democratic Republic (GDR), that facilitated and encouraged women to enter the work force. Moreover, time is a factor on many levels as well: Period effects in studies carried out in western industrialized countries show a liberalization in more recent years and younger birth cohorts seem to be more egalitarian than older ones (Baier, 2014; Mays, 2012; Brewster and Padavic, 2000; Bolzendahl and Myers, 2004; Braun et al., 1994; Endrikat, 2003; Hofäcker and Lück, 2004), though there is evidence for a return to more traditional attitudes in younger cohorts, at least in East Germany (Heller et al., 2024).

A negative correlation between education and traditional gender role concepts or sexist attitudes has been demonstrated several times (Thornton et al., 1983; Davis and Robinson, 1991; Rhodebeck, 1996; Bryant, 2003; Bolzendahl and Myers, 2004). However, according to Bolzendahl and Myers (2004), maternal employment, contrary to expectations, did not lead to a reduction in gender role sexist attitudes among women. Perceptions of economic deprivation also showed to be irrelevant for women’s gender role orientation (ibid.). This corresponds with other studies showing mixed and inconclusive results regarding the effect of the individual economic situation on political attitudes (Rippl and Baier, 2005).

Thornton et al. (1983) and Glick et al. (2002) empirically demonstrated that highly religious people tend to hold more conventional gender role views. Mays (2012) could only find a weak effect for the relationship between religiosity and sexist attitudes, although this could also be a consequence of their operationalization of religiosity, which was surveyed exclusively via churchgoing frequency.

Stober (1995) constructed a 20-item, balanced scale to measure antifeminist attitudes specifically. It was validated using a sample consisting of 102 US citizen. The scores showed high reliability, factorial validity as well as high correlations with related constructs (RWA, sexism, and openness to experience). Contrary to expectations, no differences between men and women were observed.

Simon and Kohl (2023) recently developed a scale on anti-egalitarian beliefs consisting of two subscales with five items each, antifeminism and naturalization of gender differences. Confirmatory factor analysis yielded a good model fit and structural equation models show moderate positive relationships to related phenomena (i.e., belief in a just world, right-wing authoritarianism, social dominance orientation).

Based on these results as well as the theoretical considerations outlined above, we expect antifeminism to show moderate to high correlations to gender role sexism, authoritarianism as well as right-wing extremist attitudes. Furthermore, we expect antifeminism to be higher among male participants, participants with a lower level of educational attainment, and older participants. Unlike gender role sexism, we expect values to be higher in participants living in East Germany, as we conceptualize antifeminism to be closer to authoritarianism than gender role sexism; authoritarianism, like right-wing extremism, is generally more prevalent in the East German population today. We do not expect differences with regards to income. As results regarding religious affiliation have been inconclusive, we are only investigating this on an exploratory basis. Furthermore, we are going to test a central precondition of latent mean comparisons before investigating differences based on sociodemographic variables, namely that of measurement invariance. *Measurement invariance* describes the ability of a scale to capture a certain construct equally well in all groups under investigation. Results and interpretation as well as comparison of mean scores might be heavily biased, if measurement invariance is not established (Meredith and Millsap, 1992; Van de Vijver, 2018).

### 1.3 Objective of the study

In this study, we set out to validate the *Leipzig Antifeminism Short Scale* (LAF-S), an instrument to examine antifeminism and its spread where it is not visible in organized form, but still manifest in attitudes: in different groups in the middle of society. Our analysis is an extension of the work by Höcker et al. (2020): using more advanced statistical methods, we aim to introduce the first valid and reliable short scale for antifeminism to an international readership. The scale itself is designed to monitor antifeminist tendencies efficiently in large-scale, general population samples. By testing measurement invariance and then comparing latent mean scores across different sociodemographic groups and social strata (gender, age, education, income, religious affiliation, and region of residence), we start to shed light on the distribution of antifeminist beliefs and thus lay a foundation to explore the dynamics that cause these kinds of antidemocratic attitudes.

## 2 Materials and methods

### 2.1 Participants

The sample was taken from a national, biennial representative survey conducted by the University of Leipzig, Germany, the ‘Leipzig Studies on Authoritarianism’ [former Leipzig ‘Mitte’ (Centre) Studies]. The study is designed to monitor political positions, opinions and attitudes in the German general population since 2002. Data collection for this particular study was realized by an independent institute for opinion (USUMA) from May to June of 2020. A multi-stage randomization procedure following the ADM-sampling method was performed to ensure representativeness of the sample. In 2020, 5,418 households were contacted using 258 sampling points throughout Germany. Of these households, 5,389 were eligible to participate, i.e., non-vacant and with at least one inhabitant meeting the inclusion criteria (at least 14 years of age and a sufficient ability to understand spoken and written German). In case of multi-person households, Kish selection grid was used to randomly select the target person. All participants provided informed consent; in case of minors, at least one next of kin, caretaker, or guardian provided additional consent. Sociodemographic information was obtained in a face-to-face setting. The participants were then handed a questionnaire to fill out on their own with the interviewer still present to answer any queries. Response rate was at 46.8%, leading to a total sample size consisted of *N* = 2,503. As missing values did not exceed 5% on any single item (Schafer and Graham, 2002), participants with missing values in the questionnaire were excluded for the analyses (*n* = 43). Table 1 gives an overview of the sociodemographic characteristics of the sample. When comparing sex, education, and age groups, to data provided by the Federal Statistical Office of Germany (2020) only minor deviations could be observed, including a slight overrepresentation of female and older participants as well as an underrepresentation of participants with a low level of educational attainment. Keeping these deviations in mind, we consider the sample to be representative of the German population. Thus, no weights were applied in the analysis.^2^

**Table 1:** Sociodemographic characteristics.

Variable		*n*	%	(% (Federal Statistical Office of Germany, 2020))
Sex				
	Male	1,148	46.67	49.34
	Female	1,311	53.29	50.66
	Other	1	0.04	
Age (mean; SD)		46.02	17.73	
Age group				
	14–24	370	15.04	10.00
	25–34	395	16.06	12.79
	35–44	374	15.20	12.45
	45–54	464	18.86	14.29
	55–64	441	17.93	15.16
	65+	416	16.91	21.00^a^
Place of residence				
	West	1963	79.80	
	East	497	20.20	
Education				
	<10 years	558	22.68	30.28
	=10 years	991	40.28	30.87
	>10 years	832	33.82	35.24
	Still in school	72	2.93	3.48
	Missing	7	0.28	Other: 0.08
Household equivalence income per month				
	≤1,000 EUR	337	13.70	
	>1,000 EUR–2,000 EUR	928	37.72	
	>2,000 EUR–3,000 EUR	567	23.05	
	>3,000 EUR	453	18.41	
	Missing	175	7.11	
Religious affiliation				
	Catholic church	681	27.68	
	Protestant church	631	25.65	
	Islamic religious communities	113	4.59	
	Other (Jewish, Buddhist, other Christian affiliations and more)	98	3.99	
	No religions affiliation	891	36.22	
	Missing	46	1.87	

a The share missing to 100% was <14 years of age.

### 2.2 Measures

The items for the *Leipzig Antifeminism Short Scale* (LAF-S) were taken from an 11-item antifeminism scale with three dimensions: *antifeminism*, *gender role sexism*, and *pro*-*feminism*. In this study, we focus on the *antifeminism* subdimension of the scale, as it captures a distinctly political dimension with clear connections to other antimodern resentments. It consists of four items and were developed by Charlotte Höcker, Hannah Eitel, and Oliver Decker as part of an 11-item scale with three dimensions.^3^ All items were scored on a 4-point scale ranging from 1 = “I completely disagree” to 4 = “I completely agree.” Table 2 comprises the original item wording as well as an English translation.

**Table 2:** Item wording of the *LAF-S*.

Item	English	German
AF_PO	Women often make fools of themselves in politics.	Frauen machen sich in der Politik häufig lächerlich.
AF_DE	Women who make excessive demands should not be surprised if they are put back in their place.	Frauen, die mit ihren Forderungen zu weit gehen, müssen sich nicht wundern, wenn sie wieder in ihre Schranken gewiesen werden.
AF_SE	Women often exaggerate their accounts of sexual violence to take advantage of the situation.	Frauen übertreiben ihre Schilderungen über sexualisierte Gewalt häufig, um Vorteile aus der Situation zu schlagen.
AF_HA	Feminism disrupts social harmony and order.	Durch den Feminismus werden die gesellschaftliche Harmonie und Ordnung gestört.

To establish convergent validity, a well-validated, nine-item short scale for authoritarian attitudes, the “Kurzskala Autoritarismus” [KSA-3; Beierlein et al. (2014); validated by Heller et al. (2022)] was used. It measures authoritarian attitudes based on the three dimensions proposed by Altemeyer (1981, 1996) using three items each: *authoritarian submission* as a person’s tendency to follow the rule of a strong leader, *authoritarian aggression* as the willingness to punish (socially) deviant behavior and *conventionalism* as the adherence to established rules of conduct and reluctance to change. Answers were scored using a 5-point Likert-type scale.

Subsequently, right-wing extremism was measured using the “Fragebogen zur rechtsextremen Einstellung – Leipziger Form” [FR-LF; Decker et al., 2013; validated by Heller et al. (2020)]. It is based on a definition established at a consensus conference of leading German social scientist (Kreis, 2007) and captures the following dimensions using three items each: *chauvinism, antisemitism, antifeminism, xenophobia, belittling the crimes of national-socialism,* and *agreement to a right-wing dictatorship*. A 5-point Likert-type scale is used for scoring. All item wordings in English and German may be found in the Supplementary material.

In order to establish greater generalizability of the LAF-S and to meaningfully compare latent mean scores, we seek to establish invariance across several different demographic groups, that may be found in Table 1. *Sex* was assessed in three categories: male, female, and divers. As only one person fell into the divers category, only male and female participants were included in the group comparison. Six *age groups* of roughly the same size were differentiated. Regarding the current *place of residence*, we differentiated between the states that were part of the former GDR (East Germany) and those that were not (West Germany). The districts of Berlin that used to be part of West Germany were coded accordingly. To make results more accessible, *educational attainment* was converted four different categories: low education (equivalent of less than 10 years of schooling), medium (equivalent of approx. 10 years), and high (equivalent of more than 10 years of schooling), as well as participants still in school. The latter were excluded for group comparison. *Household equivalent income* was assessed in four groups: ≤1,000 EUR, >1,000–2,000 EUR, >2,000–3,000 EUR, and >3,000 EUR. Five categories were used to differentiate *religious affiliations*: Protestant, Catholic, Muslim, and no religious affiliation as well as a residual category ‘other’ containing Jewish, Buddhist, Evangelical, Christian-Orthodox and other religious affiliations.

### 2.3 Statistical analyses

As the scale under investigation was scored with just four response categories, correct scaling is an issue. Simulation studies by Rhemtulla et al. (2012) could show that Likert-type scales with less than five response categories should be treated as ordinal instead of metric to insure correct estimation of models in (multi-group) confirmatory factor analyses [(MG)CFA]. To assess the scale’s psychometric properties, we thus first evaluated standard item statistics for ordinal data, namely median and mode. Additionally, we looked at response distributions across the four response options for each item. To make results more accessible, we also reported common indicators such as mean, standard deviation, skewness and kurtosis as well as item-total-correlation, and item difficulty. Typically, skewness and kurtosis are used as indicators for normality with a cut-off of >|2| for a nonnormal distribution (Pituch and Stevens, 2016). Item difficulty should range between 0.20 and 0.80 and item-total-correlation should be above 0.30 (Moosbrugger and Kelava, 2012). While item difficulty is not necessarily a meaningful indicator when it comes to measuring attitudes and beliefs, we include it here for comparative purposes.

We then performed confirmatory factor analysis to verify the scale’s unidimensionality. To account for the ordinal character of the data, we used the weighted least square mean and variance adjusted estimator (WLSMV) that is specifically designed for ordinal observed variables, as it does not make any assumptions about their distribution (Li, 2016).^4^ Instead of item intercepts, threshold estimates are used based on polychoric correlations. As there are no general recommendations for model fit indices and cut-off values in models with ordinal indicators yet, the following standard criteria were used in their scaled version to evaluate model fit (Schermelleh-Engel et al., 2003). *χ*^2^ and *χ*^2^ divided by the degrees of freedom should be as low as possible. For the Comparative-Fit-Index (CFI) as well as the Tucker-Lewis-Index (TLI) values >0.95 were rated as acceptable and values >0.97 as good. For the root mean square error of approximation (RMSEA) and the standardized root mean square residual (SRMR), we used a cut if of <0.05 for a good fit and <0.08 for an acceptable fit. Finally, factor loadings for each item as well as three indicators for internal consistency were calculated: Regular Cronbach’s Alpha that is based on Pearson correlations, ordinal alpha, that uses polychoric correlations instead (Zumbo et al., 2007) and McDonald’s omega (McDonald, 1999). Convergent validity is assessed by calculation correlations to right-wing extremism and its sub-dimensions as well as authoritarianism.

To establish measurement invariance, traditional approaches use MGCFA on increasingly restrictive, nested models. Following recommendations by Svetina et al. (2020) on invariance testing for ordinal data, three types of models were compared. (1) a *configural model* with no restrictions imposed, (2) a *threshold model* in which item thresholds were set equal across groups, (3) a final model in which both thresholds and loadings were equal between groups^5^. For each step, the decline in model fit indices is reported to assess whether invariance holds. Once again, no common cut-off values exist for ordinal data, as they depend on multiple factors like the number of groups, the number of factors or the number of observations per group. We thus focused on changes in the CFI, as the *χ*^2^-difference-test is known to be highly sensitive in large samples (ΔCFI should be <0.01; Cheung and Rensvold, 2002). Changes in *χ*^2^-values as well as RMSEA are reported as well.

If invariance may be assumed in the final model, latent mean scores can be meaningfully compared. To this end, the latent mean is set to zero in one of the groups in the final model, and latent means of the other groups are calculated relative to that reference group (Wu and Estabrook, 2016). Latent mean deviations from the reference group are then tested for significance using Wald *t*-test. *p*-values and standard errors are reported for each comparison. As it is still common practice in most psychological and social science research to compare observed sum or mean scores instead of latent means, we will additionally be reporting and comparing observed mean scores using ANOVA and *post hoc* TukeyHSD tests, to see if this may impact the results.^6^

RStudio 2025.05.1 Build 513 with R version 4.4.1 (2024-06-14 ucrt) was used for all analysis. Lavaan version 0.6–7 was used for (MG)CFA and latent mean comparison.

## 3 Results

### 3.1 Descriptive item statistics

Table 3 shows descriptive item statistics of the LAF-S’s four items. Mean scores were relatively low with standard deviations indicating a possible bottom effect. Yet, both skewness and kurtosis fell under the cut-off of <|2| (Pituch and Stevens, 2016), indicating a normal distribution. Item difficulty fell in the medium level, ranging from 0.42 to 0.47 and item total correlation can be judged as excellent, exceeding the cut-off of 0.40 by far (Moosbrugger and Kelava, 2012).

**Table 3:** Psychometric properties of the LAF-S.

Item	Median	Mode	Response distribution (%)^a^				Mean	Standard deviation	Skewness	Kurtosis	Item difficulty	Item total correlation
			1	2	3	4						
AF_PO	1	1	51.26	33.82	12.32	2.60	1.66	0.79	0.99	0.25	0.42	0.60
AF_DE	2	1	45.04	28.50	21.10	5.37	1.87	0.93	0.67	−0.61	0.47	0.66
AF_SE	2	1	45.65	37.24	13.62	3.50	1.75	0.82	0.87	0.06	0.44	0.59
AF_HA	2	1	47.48	34.67	14.80	3.05	1.74	0.82	0.86	−0.09	0.43	0.63

a Values over 100 are due to rounding.

### 3.2 CFA and reliability

Table 4 depicts the model fit indices of the CFA. It is visible that all indices stay within their respective cut-offs, with only the RMSEA exceeding the 0.08 mark by far. Kenny et al. (2015) could show that RMSEA tends to be high in models with only small degrees of freedom as this one. Overall, model fit can thus be judged as acceptable.

**Table 4:** Model fit indices of the CFA.

Estimator	*N*	*χ*^2^(*df*)	*χ*^2^/DF	CFI	SRMR	RMSEA (90% CI)	TLI
WLSMV	2,460	95.196 (2)	47.598	0.987	0.030	0.138 (0.115–0.162)	0.962

WLSMV, weighted least square mean and variance adjusted; *df*, degrees of freedom; *χ*^2^/*df*, minimum discrepancy, divided by its degrees of freedom; CFI, comparative-fit-index; SRMR, standardized root mean square residual; RMSEA (CI), root mean square error of approximation (confidence interval); TLI, Tucker-Lewis Index.

Table 5 shows factor loadings and internal consistency based on this estimation method. Factor loading ranged from 0.75 for Item 3 to 0.83 for Item 2. Internal consistency can be judged as good: Regular Cronbach’s alpha was at 0.81, ordinal alpha turned out a little higher at 0.86 and McDonald’s Omega was at 0.82.

**Table 5:** Standardized factor loadings as well as reliability.

Model	Standardized factor loadings				Reliability		
	Item 1	Item 2	Item 3	Item 4	Cronbach’s alpha	Ordinal alpha	McDonald’s omega
WLSMV	0.77	0.83	0.75	0.79	0.81	0.86	0.82

WLSMV, weighted least square mean and variance adjusted.

### 3.3 Convergent validity

In a first validation study by Höcker et al. (2020), convergent validity was reported by calculating Pearson correlations to adjacent constructs, namely *right-wing extremism* and *authoritarianism*. Their results are depicted in Table 6. Correlations reached from 0.27 (with *authoritarian aggression* and *authoritarian submission*) to 0.51 (with the sum score for *right-wing extremism*). These medium to high connections can be seen as an indicator of convergent validity. Regarding the related construct measuring gender role sexism, a high correlation of 0.53 was found. Correlations between gender role sexism and right-wing extremism and authoritarianism, respectively, were markedly lower than those of antifeminism with the two.

**Table 6:** Pearson correlations of the LAF-S sum score with related constructs, taken from Höcker et al. (2020).

Scale	Antifeminism	Gender role sexism
Right-wing extremism	0.51	0.39
Right-wing dictatorship	0.40	0.34
Chauvinism	0.42	0.31
Xenophobia	0.44	0.28
Antisemitism	0.44	0.38
Social Darwinism	0.44	0.38
Belittling the crimes of National Socialism	0.37	0.25
Authoritarianism	0.35	0.32
Authoritarian aggression	0.27	0.23
Authoritarian submission	0.27	0.26
Authoritarian conventionalism	0.31	0.28
Gender role sexism	0.53	–

### 3.4 Measurement invariance and comparison of latent mean scores

Results of analysis of measurement invariance may be found in Table 7. Following the cut-off of ΔCFI <0.01, invariance may be assumed in all conditions. Meaningful, i.e., unbiased, comparisons of latent mean scores across these groups are thus possible.

**Table 7:** Tests for invariance across gender, region of Germany, age group, education, income group, and religion.

	*χ* ^2^	*df*	Δ*χ*^2^ (Δ*df*)	*p*	CFI	ΔCFI	RMSEA	ΔRMSEA
Gender								
Configural	99.997	4	–	–	0.985	–	0.140	–
Thresholds	134.430	11	34.433 (7)	<0.001	0.981	0.004	0.096	0.044
Thresholds + loadings	147.971	14	13.541 (3)	<0.001	0.980	0.001	0.088	0.008
Region of Germany								
Configural	90.822	4	–	–	0.988	–	0.133	–
Thresholds	104.635	11	13.813 (7)	0.055	0.988	<0.001	0.083	0.050
Thresholds + loadings	110.670	14	6.035 (3)	0.110	0.987	0.001	0.091	0.008
Age groups								
Configural	105.765	12	–	–	0.988	–	0.138	–
Thresholds	160.608	47	54.843 (35)	<0.05	0.986	0.002	0.077	0.061
Thresholds + loadings	192.424	62	31.816 (15)	<0.01	0.984	0.002	0.072	0.005
Education								
Configural	115.919	6	–	–	0.984	–	0.152	–
Thresholds	138.756	20	22.837 (14)	0.063	0.982	0.002	0.087	0.065
Thresholds + loadings	153.927	26	15.171 (6)	<0.05	0.981	0.001	0.079	0.008
Income								
Configural	102.571	8	–	–	0.986	–	0.144	–
Thresholds	127.427	29	24.856 (21)	0.253	0.986	<0.001	0.077	0.067
Thresholds + loadings	119.793	38	−7.634 (9)^a^	1	0.988	0.002	0.061	0.016
Religion								
Configural	93.598	10	–		0.989	–	0.132	–
Thresholds	113.859	38	20.261 (28)	0.855	0.990	0.001	0.064	0.068
Thresholds + loadings	112.822	50	−1.037 (12)	<0.001	0.992	0.002	0.051	0.013

*df*, degrees of freedom; CFI, Comparative-Fit-Index; RMSEA, root mean square error of approximation.

a Due to the scaling factor in ordinal data, it is possible for the chi squared value to decrease in the difference test.

Results of the comparisons of latent mean deviations relative to a set reference group as well as comparisons of observed mean scores using ANOVA and *post hoc* TukeyHSD tests can be found in Table 8. Male participants showed significantly higher latent mean scores in antifeminism than female participants. Participants living in the former eastern German states scored higher than those in the former West. A gradient was observed regarding the educational background. Those participants with low educational attainment showed the highest endorsement of antifeminism—significantly higher than the reference group a medium level of educational attainment. Those participants with higher education on other hand exhibited significantly lower values than the reference group. No significant differences were found with regard to age groups. Regarding income, no differences between the group with an income of up to 1,000 EUR and the group with more than 1,000–2,000 EUR or the group with more than 2,000–3,000 EUR were found. The highest income group with more than 3,000 EUR exhibited significantly lower values compared to that with less than 1,000 EUR. Finally, results regarding religious affiliation suggest highest values of antifeminism in Muslim participants. Differences between Catholic and Protestant participants were significant on a 5%-level when latent mean scores were compared, with Catholic participants showing slightly higher values. While results of comparison of observed mean scores generally pointed in the same direction with only slight differences in significance levels, these differences between Catholic and Protestant participants did not turn out to be significant in the comparison of observed mean scores.

**Table 8:** Estimated and standardized latent mean deviations based on a reference group as well as comparison of (observed) composite scores.

	Latent mean comparison				Comparison of mean scores			
	Latent mean deviation (est.)	Standard error	Latent mean deviation (Std.)	*p*-value	Mean	SD	*F*-score	*p*-value
Sex								
[Ref.: female]					1.59	0.59	*F*(1, 2,457) = 190.7	**<0.001**
Male	0.49	0.04	0.66	**<0.001**	1.95	0.70		
Place of residence								
[Ref.: West]					1.73	0.66	*F*(1, 2,458) = 12.38	**<0.001**
East	0.14	0.05	0.18	**<0.01**	1.85	0.69		
Education								
[Ref.: = 10 years]					1.82	0.68	*F*(2, 2,379) = 110.1	**<0.001**
<10 years	0.13	0.05	0.18	**<0.01**	1.92	0.70		**<0.05**
>10 years	−0.36	0.05	−0.49	**<0.001**	1.56	0.58		**<0.001**
Age groups								
[Ref: 14–24 years]					1.76	0.68	*F*(5, 2,454) = 1.295	0.263
25–34 years	−0.08	0.07	−0.11	0.236	1.72	0.66		0.95
35–44 years	−0.02	0.07	−0.03	0.727	1.74	0.67		1.00
45–54 years	−0.03	0.07	−0.04	0.685	1.71	0.65		0.84
55–64 years	0.05	0.07	0.07	0.452	1.79	0.69		1.00
≥65 years	0.09	0.06	0.13	0.166	1.80	0.66		0.97
Income								
[Ref.: ≤1,000 EUR]					1.77	0.66	*F*(3, 2,281) = 15.22	**<0.001**
>1,000–2000 EUR	0.07	0.06	0.09	0.234	1.83	0.69		0.47
>2000–3,000 EUR	−0.04	0.06	−0.05	0.565	1.74	0.67		0.90
>3,000 EUR	−0.27	0.07	−0.36	**<0.001**	1.58	0.60		**<0.001**
Religious affiliation								
[Ref.: Protestant]					1.70	0.65	*F*(4, 2,409) = 4.84	**<0.001**
Catholic	0.10	0.05	0.15	**<0.05**	1.76	0.64		0.51
Muslim	0.28	0.10	0.32	**<0.01**	1.97	0.74		**<0.001**
Other	0.15	0.12	0.16	0.204	1.87	0.77		0.125
None	0.05	0.05	0.06	0.334	1.75	0.68		0.600

WLSMV estimation was used to calculate latent mean deviations. Pairwise Wald *t*-tests were employed to compare latent means. ANOVAs and *post hoc* TukeyHSD-tests were used for the comparison of observed scores. Significant group differences are put in bold.

## 4 Discussion and limitations

In this study, we set out to validate a short scale for antifeminist attitudes, the *Leipzig Antifeminism Short Scale* with four items, analyze its measurement invariance and compare latent mean scores across different social groups. We understand antifeminism as a concept related to, but also distinct from gender role sexism as it captures the direct opposition of women’s emancipatory claims and movements. It is thus more closely related to antimodern resentments like antisemitism, authoritarianism, and right-wing extremism. In the following, we will summarize and discuss the results and limitations of the validation process as well as the substantive contribution we see in the comparison of latent mean scores.

Results of the validation study using a large, representative German sample could show that the LAS-F is indeed suitable as a large-scale monitoring instrument: Using the WLSMV estimator to account for the ordinal character of the variables, CFA confirmed the unidimensionality of the scale with all indices except the RMSEA staying below the cut-off values. High values of the RMSEA could be explained by the low number of degrees of freedom in the model (Kenny et al., 2015). While testing a scale with more items could address this problem, our aim was to provide a short measurement instrument that could be used in large-scale settings. Factor loadings ranged from 0.75 to 0.83 and all indicators pointed towards good reliability. Scalar invariance could be assumed across gender, region of Germany, age groups, educational backgrounds, income, and religious affiliation. Previous analyses of convergent validity by Höcker et al. (2020) showed medium to high correlations to adjacent constructs. In line with our conception, there was a large overlap with gender role sexism, but as hypothesized, correlations of antifeminism with right-wing extremism were higher than those of gender role sexism with right-wing extremism. This serves as evidence that antifeminism is indeed at least partially distinct from gender role sexism as it is commonly measured.

Results of latent mean comparisons further reinforce this finding: while views on gender roles are known to be more egalitarian in the former eastern German states compared to the West, antifeminism (just as authoritarianism and right-wing extremism) is significantly more prevalent there. Our study could now show that while views on gender role sexism may have been reduced by GDR policy making (while often leading to a double burden of women having to take care of both income and reproductive work at home; Becker-Schmidt, 2003), antifeminism, that we consider to be more closely connected to other antimodern resentments, is even more prevalent there. Future studies could shed led on these differences by investigating the influence of historical, biographical and present-day disparities between East and West Germans on antifeminism.

In our analysis, we were further able to show that male participants are clearly more inclined towards antifeminism than female participants. The result is not surprising and in line with findings regarding gender role sexism (Baier, 2014), thus further strengthening the validity of our scale. Contrary to our expectation, no differences regarding age was found. While this may point towards a retraditionalization observed in younger cohorts, at least in East Germany (Heller et al., 2024), this topic needs further investigation, possibly also including interaction effects with living or having been socialized in East or West Germany.

With regard to educational background, latent means in antifeminism were lower for those groups with a higher level of formal education. This effect was also documented with regard to gender role sexism and may be explained by the fact that people in higher educational institutions are more likely to move in an egalitarian atmosphere. Moreover, cognitive skills and knowledge increase with higher education, which serve to reflect prejudices and generally develop a more democratic attitude (Mays, 2012). This would tie in with early works of the psychoanalyst Else Frenkel-Brunswik (1948), who described the connection between authoritarianism, prejudice and the tendency to submit to rigid conventions and black-and-white thinking, also in relation to gender roles. With the concept of ambiguity tolerance, she named a personality variable that, in contrast to authoritarian orientation, integrates the simultaneity of contradictory information, feelings and perceptions in the same person or situation (Höcker and Niendorf, 2022). The concept of tolerance of ambiguity as well as reflective functioning and perspective taking in the context of antifeminism and gender role sexism should be further investigated.

In terms of religiosity, an effect of belonging to an Islamic denomination was demonstrated. One possible explanation for the higher approval ratings among Muslims compared to Protestants could be a higher inclination towards traditional values in general (cf. Norris and Inglehart, 2004). However, the correlation could also be explained by the effect of a dogmatic understanding of religion (Pickel, 2022), which is much more pronounced among Muslims in Germany compared to other groups (ibid., p. 175). The religiously dogmatic tend to be more rejecting, while those who are socially engaged in the church are often even more open than the population average. Another analysis showed an effect of belonging to the Islamic religious community on homophobia (Pickel et al., 2020). There is still a lack of meaningful secondary analyses that describe the connection between religiosity and gender-based prejudice in a more differentiated way.

In addition to the high mean values of Muslim participants that appeared both on the latent as well as the observed mean level, a difference between Catholic and Protestant participants was detected only on the latent level, when measurement error was controlled for. While this underlines the usefulness of latent variable modeling, it could also be a result of the pairwise Wald *t*-tests that the comparison is based on in the current approach. Approaches like the Structural After Measurement approach, that allow for a more direct comparison of latent mean scores between groups should be used to investigate the robustness of these results (Rosseel and Loh, 2024). As all other comparisons of observed means were in line with the findings on latent mean comparisons, using the observed mean as a proxy for monitoring antifeminism on a large scale using the LAF-S seems reasonable [for the value of observed scores as a proxy, see also Sijtsma et al. (2024)].

Limitations of this study regard the data format. It has to be noted that the basis for this analysis, though representative, was a single cross-sectional, German sample. Like many forms of antimodern resentment, antifeminism is likely subject to temporal trends (Lang and Peters, 2018). Long-term, longitudinal or repeated cross-sectional analyses are needed to study trends and changes in antifeminist endorsement. Moreover, cross-cultural validity needs to be investigated. In the current study, the LAF-S was embedded in a larger set of items on gender-related attitudes. As all of them covered different gender-related topics (e.g., family life and working arrangements) and some of them were reversely coded (e.g., items on the support of feminism), we do not expect results to differ, if the LAF-S is being used on its own. Ideally, this should be checked again using an experimental setting and/or randomizing item presentation.

While results point towards a strong connection of antifeminism with both authoritarianism and right-wing extremism (see Table 6, taken from Höcker et al., 2020), further analyses of the interaction of antifeminism with social dominance, racism, and antisemitism are necessary. The paths to radicalization taken by right-wing terrorists often reveal connections to antifeminist and other inhumane ideologies, as can be seen in online communities such as the so-called “manosphere” (Barcellona, 2022). Open declarations of antifeminist ideologies can be found alongside antisemitic and racist statements, for example in the cases of the perpetrators in the attacks in Halle and Hanau in Germany 2019 and 2020. They potentially harbor an idealized nationalist and “White” image of family and women, which is to be protected against the supposed emancipative threat and against modern fragmentation. It is evident that women demanding more than what their social roles currently allow them, are most likely to be verbally and even physically attacked in several situations. What is certain is that antifeminism is part of an authoritarian reaction to social challenges of our time; it seems to offer an orientation and the hope for a harmony, but ultimately hardens and polarizes the current negotiations of the gender order to the point of raw violence (Höcker and Niendorf, 2022). Our study showed that the LAS-F can serve as an efficient measure to monitor antifeminist attitudes in order to better understand its dynamics over time and in different cultural settings as well as its connection to other forms of antimodern attitudes thus helping to eventually minimize the prevailing oppression of women’s emancipation movements.

## Data Availability

The dataset presented in this article are not readily available because it was generated as a joint project of several different universities. Due to missing consent of all parties involved, we are unable to make the dataset publicly available. The parts supporting the findings of this study will be provided by the corresponding author upon reasonable request. Requests to access the datasets should be directed to ayline.heller@gesis.org.

## Footnotes

^1^The völkisch movement was a German nationalist protest movement with an initial boom in the late 19th century. It strove for an anti-egalitarian, militaristic, male-centered society and was also predominantly male, protestant, bourgeois and middle-class in its composition and goals. The völkisch worldview was fundamentally antisemitic and racist and based on images of a homogenous ethnic body and nationalist romanticism.

^2^Applying individual-level weights to account for respondents’ chance of selection due to household size did not slightly affected results of the CFA in a meaningful way.

^3^The scale developed by Höcker, Eitel, and Decker, measures explicit and subtle antifeminist attitudes, based on items drawn from exiting social research. Items were presented to several expert groups to ensure content validity. For more information on scale construction and item selection, see Höcker et al. (2020).

^4^While using robust maximum-likelihood estimation in the CFA did not have a meaningful impact on model fit, we decided to use WLSMV estimation as it is more appropriate giving the ordinal character of the response scale.

^5^While invariance testing for continuous variables typically includes constraining intercepts to be equal across groups, this type of constraint is not necessary for ordinal indicators. In fact, a model with thresholds, loadings and intercepts constraint leads to the same results as (3).

^6^Wilcox- and Kruskal-Wallis-tests with *post hoc* Dunn’s test for group comparisons were also applied. All results pointed in the same direction with the same levels of significance as in the ANOVAs.
