# Supplementary material for: Assessing antifeminism introducing the Leipzig Antifeminism Short Scale (LAF-S) in a German representative sample
Source: Front Psychol. 2025 Dec 3;16:1596397. doi: 10.3389/fpsyg.2025.1596397 (PMC12709201; doi:10.3389/fpsyg.2025.1596397)
Supplement: Supplementary file 1 [file Table_1.DOCX]

Supplementary Material

# Supplementary Table

| **Supplementary Table 1**. Original wording and translation of the FR-LF | | | |
| --- | --- | --- | --- |
| **Item** | **German** | **English** | **Dimension** |
| 1 | Im nationalen Interesse ist unter bestimmten Umständen eine Diktatur die bessere Staatsform. | Under certain circumstances a dictatorship better serves the national interest. | Support for a right-wing dictatorship |
| 2 | Ohne Judenvernichtung würde man Hitler heute als großen Staatsmann ansehen. | Had it not been for the Holocaust, Hitler would be regarded as a great statesman today. | Belittling the crimes of National Socialism |
| 3 | Was Deutschland jetzt braucht, ist eine einzige starke Partei, die die Volksgemeinschaft insgesamt verkörpert. | Germany needs a strong single party that represents the ethnic community as a whole. | Support for a right-wing dictatorship |
| 4 | Wir sollten einen Führer haben, der Deutschland zum Wohle aller mit starker Hand regiert. | We should have a leader that rules Germany with a firm hand to the benefit of all. | Support for a right-wing dictatorship |
| 5 | Wie in der Natur sollte sich in der Gesellschaft immer der Stärkere durchsetzen. | Just as in nature, the strongest in a society should always get their way. | Social Darwinism |
| 6 | Die Ausländer kommen nur hierher, um unseren Sozialstaat auszunutzen. | Foreigners only come here to abuse the welfare system. | Xenophobia |
| 7 | Auch heute noch ist der Einfluss der Juden zu groß. | The influence of the Jews is still too strong. | Antisemitism |
| 8 | Wir sollten endlich wieder Mut zu einem starken Nationalgefühl haben. | We should dare to have strong nationalist feelings again. | Chauvinism |
| 9 | Eigentlich sind die Deutschen anderen Völkern von Natur aus überlegen. | The Germans are actually superior to other people in nature. | Social Darwinism |
| 10 | Wenn Arbeitsplätze knapp werden, sollte man die Ausländer wieder in ihre Heimat zurückschicken. | When jobs are scarce, foreigners should be sent home. | Xenophobia |
| 11 | Die Verbrechen des Nationalsozialismus sind in der Geschichtsschreibung weit übertrieben worden. | The crimes of National Socialism have been greatly exaggerated. | Belittling the crimes of National Socialism |
| 12 | Was unser Land heute braucht, ist ein hartes und energisches Durchsetzen deutscher Interessen gegenüber dem Ausland. | Today our country needs to firmly and energetically enforce its interests against other nations. | Chauvinism |
| 13 | Die Juden arbeiten mehr als andere Menschen mit üblen Tricks, um das zu erreichen, was sie wollen. | More than other people, the Jews use dirty tricks to achieve their goals. | Antisemitism |
| 14 | Das oberste Ziel der deutschen Politik sollte es sein, Deutschland die Macht und Geltung zu verschaffen, die ihm zusteht. | The highest aim of German politicians should be to ensure that Germany has the power and recognition it deserves. | Chauvinism |
| 15 | Es gibt wertvolles und unwertes Leben. | There is worthy and unworthy life. | Social Darwinism |
| 16 | Die Bundesrepublik ist durch die vielen Ausländer in einem gefährlichen Maß überfremdet. | Germany is losing its identity because of the large number of foreigners. | Xenophobia |
| 17 | Die Juden haben einfach etwas Besonderes und Eigentümliches an sich und passen nicht so recht zu uns. | The Jews just have something peculiar about them and don’t really fit in with us. | Antisemitism |
| 18 | Der Nationalsozialismus hatte auch seine guten Seiten. | National Socialism also had positive aspects. | Belittling the crimes of National Socialism |
